# Impact of Corticosteroids in Suprascapular Nerve Block on Pain and Function in Chronic Rotator Cuff Disease: A Retrospective, Observational, Longitudinal, Analytical Cohort Study

**DOI:** 10.3390/medsci13040252

**Published:** 2025-10-31

**Authors:** Javier Muñoz-Paz, Ana Belén Jiménez-Jiménez, Antonio Hidalgo-Jorge, María Nieves Muñoz-Alcaraz, José Peña-Amaro, Fernando Jesús Mayordomo-Riera

**Affiliations:** 1 Interlevel Clinical Management Unit of Physical Medicine and Rehabilitation, Reina Sofia University Hospital, Cordoba and Guadalquivir Health District, 14011 Cordoba, Spain; javier.munoz.paz.sspa@juntadeandalucia.es (J.M.-P.); anab.jimenez.jimenez.sspa@juntadeandalucia.es (A.B.J.-J.); sr1marif@uco.es (F.J.M.-R.); 2 Maimonides Biomedical Research Institute of Cordoba (IMIBIC), Reina Sofia University Hospital, University of Cordoba, 14004 Cordoba, Spain; 3 Department of Applied Physics, Radiology and Physical Medicine, University of Cordoba, 14004 Cordoba, Spain; 4 Faculty of Medicine and Nursing, University of Cordoba, 14004 Cordoba, Spain; h92hijoa@uco.es; 5 Department of Morphological and Socio Sanitary Sciences, University of Cordoba, 14004 Cordoba, Spain; cm1peamj@uco.es

**Keywords:** nerve block, glucocorticoids, chronic pain, rotator cuff injuries

## Abstract

**Background/Objectives**: Suprascapular nerve block (SSNB) is a useful therapeutic option for chronic shoulder pain, although the synergistic use of corticosteroids with anesthetics to prolong its effect is a controversial topic. The primary objective of this study was to compare the evolution of pain and functionality using the visual analog scale (VAS) and the Disabilities of the Arm, Shoulder and Hand (DASH) questionnaire between patients treated with SSNB with corticosteroids (cSSNB) and without them (sSSNB). **Methods**: A retrospective, observational, longitudinal, analytical cohort study was conducted in 28 patients (14 n per group) aged 50–80 years who had undergone SSNB with 4 mL of 0.25% bupivacaine and 40 mg/mL triamcinolone during 2024 for chronic shoulder pain lasting more than 6 months. The variables to be collected were VAS, DASH, range of motion (ROM) and Lattinen Index (LI) at baseline, the first and the third month. Patients were grouped according to the type of SSNB (cSSNB vs. sSSNB) and analyzed longitudinally and cross-sectionally using IBM-SPSS Statistics version 28.0.0. **Results**: Regarding pain, the cSSNB obtained a significant reduction in the median VAS of 4 points in the first month (*p* = 0.001) and in the third month (*p* = 0.002). In addition, significantly lower evaluations in VAS were obtained in the third month of 3 points (*p* = 0.04) in favor of the cSSNB. Regarding functionality, a reduction in evaluations with respect to the initial DASH were observed only in the cSSNB, with a difference in the first month of 21.80 points (*p* = 0.001) and 21.35 points (*p* = 0.003) in the third month. In addition, differences between groups were found, in favor of the cSSNB, of 19.20 points (*p* = 0.017) in the first month and 12.55 points (*p* = 0.012) in the third month. **Conclusions**: The combined use of corticosteroids in SSNB appears to be associated with better short-to medium-term outcomes in terms of pain and function, compared to the use of SSNB without corticosteroids in chronic rotator cuff pathologies.

## 1. Introduction

### 1.1. Background and Justification

Chronic shoulder pain is a pathology that significantly affects a large part of the population. Its prevalence is very heterogeneous, although when talking about global community data the values usually hover around 16% [1], making it one of the most common causes of referral to primary care [2,3]. This situation is accentuated in parallel with aging [4] and it can reach prevalences close to 26% in individuals over 70 years [1,2] with repetitive “physically demanding occupations” [5]. Estimations obtained from systematic reviews show that it is more common in women [2].

The etiology of this condition is highly diverse. However, rotator cuff tendon pathology is considered the most common cause of chronic shoulder pain [4], more so after the age of 65 [6,7] and it can cause “50–85% of shoulder pain diagnoses” [8]. More specifically, the tendon structure that concentrates most of the pathology in the rotator cuff is the supraspinatus (SS) tendon [7]. The involvement of this tendon has been linked to high-intensity, repetitive movements associated with certain occupations that require performing activities with the arms raised above the head [9].

Among other risk factors associated with the development of chronic shoulder pathologies, type 2 diabetes mellitus (DM2) stands out. Poor glycemic control, with elevated levels of glycosylated hemoglobin (HBA1c), favors a chronicity of the pathology by delaying the regeneration of tendon fibers [10]. Specifically, it has been documented that the presence of this condition can increase the probability of suffering from rotator cuff conditions by 1.5 times more compared to those patients who do not have it [11].

The clinical course and resolution of this condition show great interindividual variability. However, approximately 50% of cases resolve within six months [2]. For the remaining patients, symptoms may persist and extend over several years, resulting in considerable functional disability and therefore generating a significant impact on work, reflected in a high number of medium- and long-term sick leaves [12].

Therapeutic options for rotator cuff disease include a diverse approach based on a thorough history and physical examination to help the rehabilitation physician arrive at an accurate diagnosis. As a basic initial treatment approach, conservative measures such as cryotherapy and lifestyle modifications to avoid physical activities that may aggravate symptoms are recommended. This therapeutic level also includes various conventional rehabilitation techniques based on kinesitherapy and the administration of analgesic drugs [4].

On the other hand and as a second line of treatment, additional interventional therapeutic alternatives are considered, including suprascapular nerve block (SSNB), the technique that is the subject of this study [13,14,15].

Currently, nerve blocks represent a key tool in the field of rehabilitation, especially interventional rehabilitation. They can be effectively integrated into therapeutic strategies for pain management, providing significant relief to patients [13,16].

**Figure 1:**
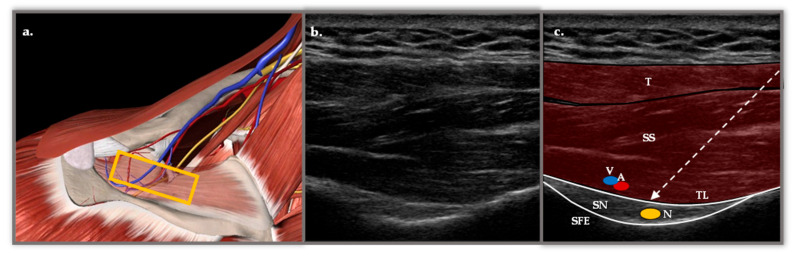
(**a**) Anatomical representation in Essential Anatomy 5 of the approach to the SSBN in the supraspinatus fossa. The muscular plane of the trapezius (T) has been removed and the supraspinatus (SS) has been faded, revealing the depth of the suprascapular nerve (N), vein (V) and artery (A) in the scapular notch (SN). The yellow rectangle represents the longitudinal position of the probe. (**b**) Longitudinal ultrasound section with a 6 and 15 MHz flat probe in supraspinatus fossa. (**c**) Representation of the most important structures with two muscle layers corresponding to the T and the SS. Below, two hyperechoic structures appear, the transverse ligament (TL) and the SE, which form the roof and floor of the fossa through which the N passes. The TL separates the V and A structures from the N. The dashed arrow represents the course of the needle with a posterior approach from medial to lateral.

The basic foundation of this technique lies in the sensory innervation of the shoulder, which accounts for almost 70% of the suprascapular nerve [15], specifically the posterior-superior area of the rotator cuff [17]. SSNB can be performed using different approaches, either based on anatomical landmarks or guided by ultrasound (US), a method that offers greater precision and safety [18]. The approach used in the Physical Medicine and Rehabilitation (PM&R) Department of the Reina Sofía University Hospital (HURS) is the posterior medial-to-lateral approach with US. Figure 1.

The therapeutic indications for this type of block range from acute pain to chronic conditions. In this context, a study by Mardani-Kivi et al. concluded that SSNB was “an effective therapy, providing long-term pain relief and improving shoulder joint mobility in patients with adhesive capsulitis” [19]. Likewise, in hemiplegic patients suffering from chronic shoulder pain, SSNB was found to be an effective therapeutic option, with a significant reduction in pain levels according to the visual analgesic scale (VAS) [20].

Despite being an increasingly common technique in the clinical practice of PM&R, there are still many open fronts and unclarified issues that allow this technique to be developed. Generally, SSNB is performed by injecting a local anesthetic at the physician’s choice, usually bupivacaine, mepivacaine, or ropivacaine at concentrations of 0.25–0.5%. Adjuvant drugs, such as corticosteroids, may or may not be added [21]. This combination is based on the idea of prolonging the useful life of SSNB. In the PM&R service at HURS, the technique typically used involves 4 mL of 0.25% bupivacaine with 40 mg/mL triamcinolone.

Currently, the exact mechanism of synergistic action by which the use of corticosteroids in combination with anesthetic prolongs their effect is not fully understood. Pain reduction is considered to be linked to the interaction of corticosteroids with nociceptive C fibers, mediated through glucocorticoid receptors and inhibitory potassium channels [22]. Several studies have shown that perineural administration of corticosteroids significantly contributes to the reduction in ectopic discharges associated with pain [23] in addition to the inhibitory effect it has on nitric oxide [24].

Previous studies have evaluated the clinical efficacy of dexamethasone combined with other drugs in the peripheral nerve blockade during surgical procedures, with the aim of prolonging the duration of the anesthetic effect in the postoperative period. In particular, one conducted in 2017 showed that the use of dexamethasone in perineural blocks in a surgical setting generated significant results, prolonging the duration of the analgesic effect between 6 and 7 h. In addition, a considerable decrease in the intensity of postoperative pain was observed, which was maintained at 12 and 24 h after the intervention [25]. Contrary to this study, it was concluded that although the efficacy of perineural dexamethasone nerve block is superior to its non-use, this does not represent a statistically significant difference [26].

The truth is that these types of studies focus on determining the effect of this combination in the very short term, over periods of hours or days and do not focus on finding the clinically significant effect it could have in the medium to long term.

Whether or not to combine corticosteroids is also a matter of debate, due to the “risk/benefit balance” of the adverse effects that may occur. The use of corticosteroids has been linked to local effects such as “rashes, hypopigmentation and atrophy of the skin, infection, tendon rupture, accelerated progression of osteoarthritis and bone damage” or systemic effects that may include “adrenal insufficiency, facial flushing, hypertension, hyperglycemia and osteoporosis” [27].

For this reason, the use of corticosteroids in hypertensive or diabetic patients, which is very common in today’s population, can alter the patient’s baseline balance. Specifically, it can increase “systolic blood pressure by up to 15 mm Hg in 24 h” [28]. It can also produce significant transient increases in blood glucose levels in patients with DM who received injected corticosteroids for the treatment of their shoulder pain [29].

To the possible side effects of corticosteroids, one must also add the possibility of systemic toxicity that local anesthetic agents have [30]. The use of bupivacaine and the increase in concentration has been related to cardiotoxic effects, due to the possible blockage of sodium channels [31].

Given this background, it is necessary to evaluate the different effects of concomitant or non-concomitant use of corticosteroids on the SSNB in chronic shoulder pathology.

### 1.2. Initial Hypothesis

Patients with chronic shoulder pain who received corticosteroid-containing SSBN (cSSNB) achieved VAS and Disabilities of the Arm, Shoulder and Hand (DASH) scores at the first and third month of treatment similar to patients who received non-corticosteroid SSBN (sSSNB) in the population.

### 1.3. Objectives

The objective of the study was to compare whether the reduction in VAS scores and improvement in functionality measured by the DASH scale at one and three months in patients with chronic shoulder pain receiving the cSSNB were equal to those in patients receiving the sSSNB.

Additionally, the following secondary objectives were established:

- To establish differences in range of motion (ROM) between the two treatment groups.
- To assess the impact of both treatments on LI scores and oral analgesic use.

## 2. Materials and Methods

### 2.1. Study Design—Setting

A retrospective observational longitudinal analytical cohort study was carried out in patients from the PM&R service of HURS who were diagnosed with chronic shoulder pain, due to various pathologies related to the rotator cuff and who underwent SSNB at the initial consultation with 4 mL of 0.25% bupivacaine and 40 mg/mL triamcinolone, during the year 2024. The STROBE guideline [32] was followed to ensure compliance with the methodological and ethical requirements.

**Figure 2:**
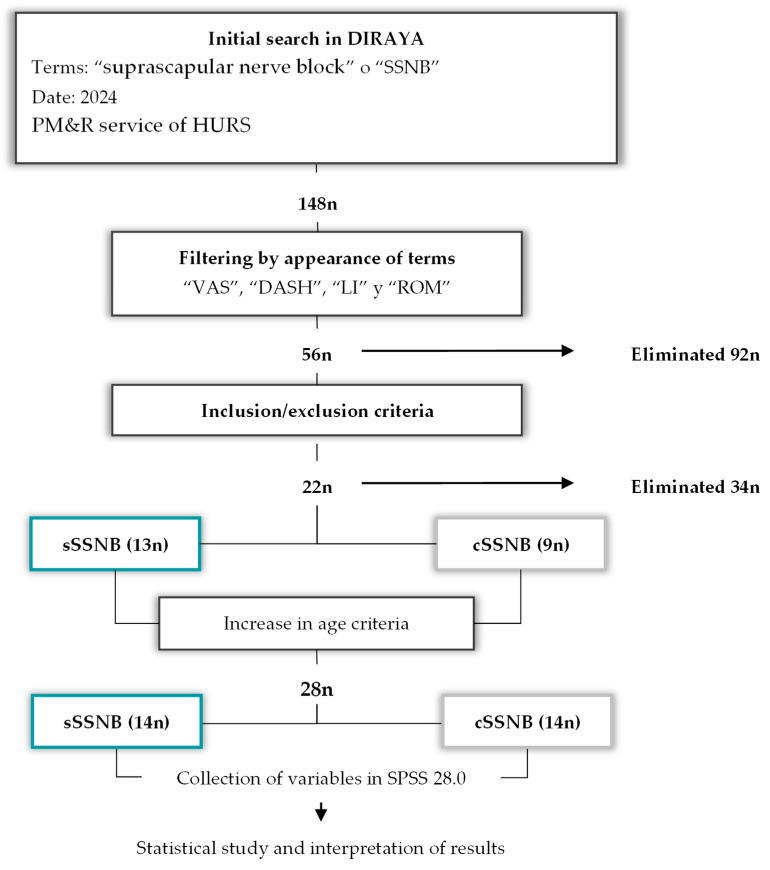
Diagram of the patient selection process (n) according to key terms and inclusion/exclusion criteria, as well as the final process of collecting variables, creating databases and interpreting them.

An initial search was conducted in the Andalusian Health Service’s healthcare information and management system (DIRAYA) using the terms “suprascapular nerve block” or “SSNB,” yielding a total of 148 patients. These results were filtered using the terms “VAS” “DASH,” “Lattinen,” and “ROM” in clinical reports and patients with “missing data” were eliminated. A total of 56 patients were obtained. After applying inclusion and exclusion criteria, 22 patients remained: 9 treated with cSSNB and 13 with sSSNB. Since the “n” value calculated for the sample size (Section 2.5) was not sufficient, it was decided to extend the age limit from 70 years to 80 years, yielding a total of 28 patients, divided into two equal groups, according to the treatment applied. See Figure 2.

### 2.2. Participants—Selection Criteria

- Inclusion Criteria:
  - Age ≥ 50 and ≤80 years.
  - Shoulder pain lasting more than 6 months.
  - Resistance to first-line treatments (oral medication, physical therapy, or injections).
  - The following data must be completed: VAS, DASH, ROM, Lattinen Index (LI), Patient Global Impression of Improvement (PGI-I) and Clinician Global Impression of Improvement (CGI-GI) scales.
- Exclusion Criteria:
  - Missing data of the patient when data were collected in the database.
  - Failure to complete the appropriate informed consent form for joint or periarticular injections.

### 2.3. Data Sources/Measurement

- Initial VAS (inVAS), at 1 month (1mVAS) and at 3 months (3mVAS) [33,34,35].

The VAS is a validated tool that allows for subjective measurement of acute and chronic pain intensity. The scale consists of a 10 cm horizontal line, with both ends representing extreme levels of pain. On the left is the complete absence of pain and on the right is the equivalent of the maximum intensity. It is measured in points and a reduction of around 2 points on the VAS is generally considered to represent a minimal clinically important change (MCID).

- Initial DASH (inDASH), at 1 month (1mDASH) and at 3 months (3mDASH) [36,37].

The DASH questionnaire is designed to assess functionality related to upper limb pathologies. The questionnaire consists of 30 questions related to symptoms, functional capacity and quality of life, scored from 1 to 5 depending on the difficulty of performing each task. It is measured in points and a 12-point variation is generally considered an MCID.

- ROM. Flexion (FLEX), abduction (ABD), external rotation (ER) and internal rotation (IR) [1,33].

ROM assessment, measured in degrees (°), is a fundamental practice in the analysis of shoulder pathologies. It is important to measure ROM both actively and passively to rule out various conditions, such as adhesive capsulitis or rotator cuff tendinopathy.

In this context, ROM assessment allows the determination of the capacity of FLEX, ABD and RE with the shoulder at 90° of abduction and IR in the same posture.

As a rule, 180° is considered the norm for ABD and FLEX and 90° for RE and IR [38]. However, these values often vary depending on multiple variables, which is why functionality is usually measured with scales like the one used in this study. This means that, to date, the MCID for ROM is not well-established due to the variety of pathologies that can be associated with the rotator cuff.

- Initial LI (inLI), at 1 month (1mLI) and at 3 months (3mLI) [39,40].

The LI test is a validated tool for measuring pain, especially in patients with chronic conditions. This test assesses five different aspects, awarding scores between zero and four. Of these aspects, the evaluation of the reduction in analgesic consumption is particularly interesting:

1. Pain intensity.
2. Pain frequency.
3. Painkiller use.
  ○ Does not take (0 points)
  ○ Occasionally (1 point)
  ○ A few regularly (2 points)
  ○ Many regularly (3 points)
  ○ Many very frequently (4 points)
4. Degree of disability.
5. Hours of sleep.

- PGI-I [41]

The PGI-I scale is not validated for this condition, but it is very useful for assessing the patient’s perception of their improvement or deterioration after receiving treatment. The scale consists of a single question in which the patient rates the relief experienced from 1 to 7:

1. Very much better 2. Much better 3. A little better 4. No change 5. A little worse 6. Much worse 7. Very much worse.

Scores of “Very much better” or “Much better” are usually considered positive clinical differences.

- CGI-GI [41].

The CGI-GI scale is used to globally assess a patient’s improvement over time, as judged by the healthcare professional, although it is not validated for this pathology. The degree of improvement or deterioration is reflected on a scale ranging from 1 to 7:

1. Very much better 2. Much better 3. A little better 4. No change 5. A little worse 6. Much worse 7. Very much worse.

Scores of “Very much better” or “Much better” are usually considered positive clinical differences.

Although PGI-I and CGI-GI are not validated scales, their use as a secondary variable would provide important information regarding the point of view of the treatment results from the perspective of the patient and the doctor. In addition, the following variables were collected and taken into account: age (years), sex (male/female), affected shoulder (right/left), concomitant diseases (arterial hypertension (HTA)/DM) and previous treatments (oral medication, manual therapy, corticosteroid infiltrations, platelet-rich plasma or nothing).

Since this was a retrospective study, the data collected could not be extended beyond 3 months since the service’s internal protocol requires reviews at 1 and 3 months.

### 2.4. Bias

This study is not blinded.

### 2.5. Sample Size

The GRANMO calculator [42,43,44,45] was used to calculate the sample size. Considering a statistical significance level of 0.05 and a statistical power greater than 80% in a bilateral contrast, it was calculated that 11 participants in the sSSNB group and 11 in the cSSNB group were required to detect a difference equal to or greater than 1 unit in VAS. A common standard deviation of 0.8 was assumed using similar studies as a reference [19]. Furthermore, a loss to follow-up rate of 0% was estimated.

### 2.6. Statistical Methods

Statistical analysis was performed using IBM-SPSS Statistics version 28.0.0. Firstly, a general descriptive study of the previously described baseline variables was conducted, as shown in Table 1.

**Figure 3:**
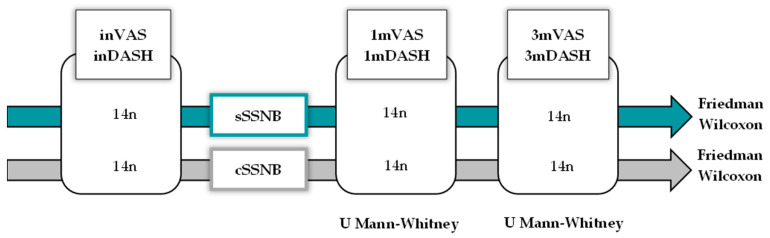
Diagram representing the statistical method used to analyze the VAS and DASH variables, both cross-sectionally at three points in time (initial, 1 month and 3 months) for the SSNB using a U Mann–Whitney test and longitudinally using the Friedman and Wilcoxon tests.

Given the small sample size, nonparametric tests were used for inferential statistics. The Mann–Whitney U test was used for cross-sectional studies and the Friedman and Wilcoxon tests were used for longitudinal and post hoc studies. All contrasts were two-tailed and those with *p* < 0.05 were considered significant. Post hoc test results show exploratory data. See Figure 3.

The following results are presented:

- Quantitative variables: median measurement [IQR].
- Qualitative variables-n (%) measurement.
- Variables with significant differences: median measurement [IQR] (statistic; significance).

## 3. Results

### 3.1. Descriptive Data

**Figure 4:**
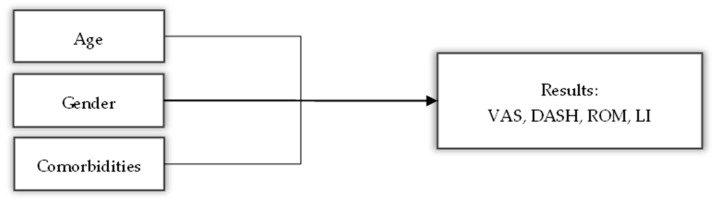
Dynamic acyclic graph showing the possible influence of variables such as age, gender and comorbidities on the results of the main variables.

The descriptive results for all variables and the statistical tests for the baseline variables are presented in Table 1. A directed acyclic graph (DAG) was also created (Figure 4) to show how certain variables can act as confounders when analyzing the results of this study.

Age, gender and comorbidity are known as common influencing factors in chronic pain [46]. Age and comorbidities are variables that go hand in hand due to their association with the degeneration of type C and Aβ somatosensory fibers responsible for detecting pain [47]. Regarding gender, “women reported higher levels of pain intensity and greater pain-related disability than men” [46].

The sample consisted of 28 patients, 14 in each group. The median age was 61.00 years [53.0–66.0] in the sSSNB group and 63.00 years [58.5–70.0] in the cSSNB group. The sex distribution was identical in both groups, with 11 (78.6%) women and 3 (21.4%) men. Regarding comorbidities, five (35.7%) of the patients in each group had HTA; two (14.3%) patients in the sSSNB group vs. three (21.4%) patients in the cSSNB group had DM. The proportion of patients without comorbidities was five (50%) patients in the sSSNB group and four (42.9%) patients in the cSSNB group. Regarding prior treatments, injections were the most common treatment, with seven (50%) injections in sSSNB vs. eight (57.1%) injections in cSSNB, followed by other isolated techniques such as SSNB blockade, physical therapy, or PRP, with low representation in both groups. There were no differences between groups regarding age, gender, comorbidities, or prior treatments.

Regarding baseline clinical variables, VAS pain scores were comparable between groups. sSSNB had 8.50 points [7.75–9.25] vs. 8.00 points [7.75–9.25] in cSSNB. DASH disability scores were also similar at baseline: sSSNB had 63.50 points [50.75–80.00] and cSSNB had 62.60 points [34.25–66.43].

Regarding range of motion, there were no differences between the ROMs at baseline. The inFLEX was 125.00° [90.00–180.00] in sSSNB and 165.00° [90.00–180.00] in cSSNB. The inABD showed values of 125.00° [90.00–180.00] in sSSNB and 137.50° [90.00–180.00] in cSSNB. The inIR was 90.00° [77.50–90.00] in sSSNB and 90.00° [75.00–90.00] in cSSNB. The inER was 90.00° [72.50–90.00] in sSSNB and 85.00° [43.75–90.00] in cSSNB.

In inLI, scores were similar, with 13.50 points [8.75–15.25] in sSSNB and 12.00 points [10.50–14.25] in cSSNB. Finally, analgesia scores were also comparable in both groups at baseline, with 3.00 points [1.75–3.00] in sSSNB and 3.00 points [1.75–3.00] in cSSNB.

The PGI-I results in the group treated with sSSNB were as follows: “very much better” in one (7.1%) patient, “much better” in three (21.4%) patients and “a little better” in eight (57.1%) patients. The results “no change” or “a little worse” represented one (7.1%) patient, respectively; in the group treated with cSEBNs, the following were obtained: “very much better” in five (35.7%) patients, “much better” in five (35.7%) patients and “a little better” in four (28.6%) patients.

The CGI-GI yielded the following results in those treated with sSSNB: “very much better” in three (21.4%) patients, “much better” in four (28.8%) patients, “a little better” in four (28.8%) patients, “no change” in one (7.1%) patient and “a little worse” in two (14.3%) patients. In the group that received cSSNB, “very much better” was noted in nine (64.3%) patients, “much better” in three (21.4%) patients and “a little better” in two (14.3%) patients.

### 3.2. Main Results

- VAS

**Figure 5:**
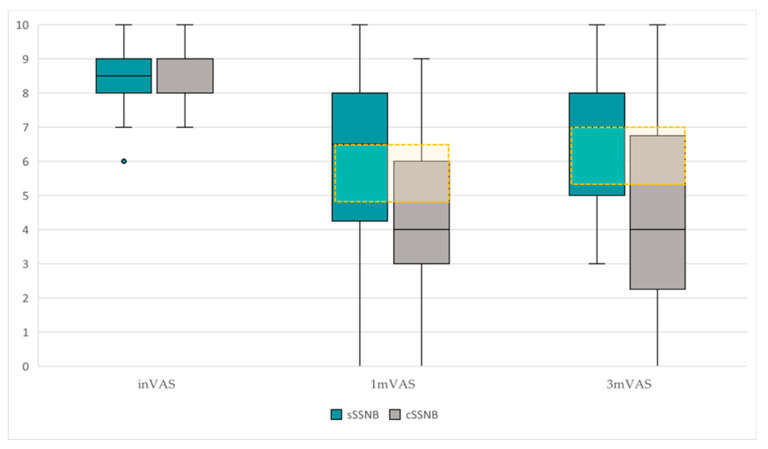
Comparative boxplot of the dispersion and VAS scores between groups, sSSNB (blue) vs. cSSNB (gray), at initial, first and third months. The yellow box represents the MCID.

The results obtained from VAS, presented descriptively in Table 1, are represented in Figure 5 and they show the following statistical results:

The longitudinal comparative study (Table 2) between inVAS, 1mVAS and 3mVAS scores, according to treatment groups, reveals the following data:

- In the sSSNB group, significant differences were found over time (χ^2^ = 14.80, *p* = 0.001). Post hoc studies demonstrated that the differences were between inVAS vs. 1mVAS (8.50 points [7.75–9.25] vs. 6.50 points [3.75–8.00]) (Z = −2.95; *p* = 0.003) and between inVAS vs. 3mVAS (8.50 points [7.75–9.25] vs. 7.00 points [5.00–8.25]) (Z = −2.85; *p* = 0.004). There were no significant differences in 1mVAS vs. 3mVAS.
- Significant differences were also found over time in the cSSNB group (χ^2^ 19.96; *p* = 0.001). Post hoc studies showed that the differences were between inVAS vs. 1mVAS (8.00 points [7.75–9.25] vs. 4.00 points [2.75–6.25]) (Z = −3.19; *p* = 0.001) and between inVAS vs. 3mVAS (8.00 points [7.75–9.25] vs. 4.00 points [2.00–7.00]) (Z = −3.07; *p* = 0.002). There were no significant differences in 1mVAS vs. 3mVAS.

The cross-sectional study (Table 1) between groups (sSSNB vs. cSSNB), in 1mVAS and 3mVAS, showed the following results:

- The cSSNB group obtained significantly lower VAS scores than the sSSNB group in 3mVAS (7.00 points [5.00–8.25] vs. 4.00 points [2.00–7.00]) (U = 53.50; *p* = 0.04). In 1mVAS, no differences were found between groups.

- DASH

**Figure 6:**
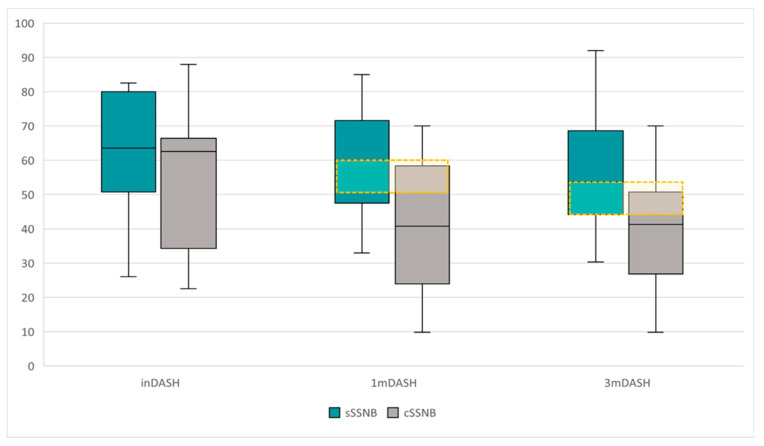
Comparative boxplot of the dispersion and DASH scores between groups, sSSNB (blue) vs. cSSNB (gray), at initial, first and third months. The yellow box represents the MCID.

The results obtained from DASH, presented descriptively in Table 1, are represented in Figure 6 and they showed the following statistical results:

The longitudinal comparative study (Table 2) between inDASH, 1mDASH and 3mDASH scores by treatment group reveals the following data:

- No significant differences were found over time in the sSSNB group.
- Significant differences were found over time in the cSSNB group (χ^2^ 19.74; *p* < 0.001). Post hoc studies showed that the differences were between inDASH vs. 1mDASH (62.6 points [34.25–66.43] vs. 40.80 points [23.95–58.33]) (Z = −3.30; *p* = 0.001) and between inVAS vs. 3mVAS (62.6 points [34.25–66.43] vs. 41.25 points [26.83–50.71]) (Z = −3.01; *p* = 0.003). There were no significant differences in 1mDASH vs. 3mDASH.

The cross-sectional study (Table 1) between groups (sSSNB vs. cSSNB), in 1mDASH and 3mDASH, obtained the following results:

- The cSSNB group obtained significantly lower scores than the sSSNB group both in 1mDASH (60.00 points [47.50–71.56] vs. 40.80 points [23.95–58.33]) (U = 46.00, *p* = 0.017) and in 3mDASH (53.80 points [44.18–68.58] vs. 41.25 points [26.83–50.71]) (U = 43.50; *p* = 0.012).

### 3.3. Analisis Secundarios

- ROM

The results obtained from the ROMs, presented descriptively in Table 1, yield the following statistical results.

In the longitudinal study (Table 2), significant differences were found only in patients treated with cSSNB. In FLEX (χ^2^ = 13.15, *p* = 0.001), differences were found between inFLEX–1mFLEX (165.00 ° [90.00–180.00] vs. 180.00° [150.00–1800]) (Z −2.38; *p* = 0.018) and in inFLEX–3mFLEX (165.00° [90.00–180.00] vs. 180.00° [157.50–180.00]) (Z = −2.53; *p* = 0.012). In the ABD (χ^2^ = 15.10; *p* = 0.001), differences were found between inABD– 1mABD (137.50° [90.00–180.00] vs. 180.00° [131.25–180.00]) (Z = −2.53; *p* = 0.011) and in inABD–3mABD (137.50° [90.00–180.00] vs. 180.00° [157.50–180.00]) (Z = −2.68; *p* = 0.007). No differences were found in IR, unlike in ER (χ^2^ = 7.60; *p* = 0.022), where differences were found between inRE–3mRE (85.00° [43.75–90.00] vs. 90.00° [72.50–90.00]) (Z = −2.03; *p* = 0.042).

In the cross-sectional study (Table 1) between groups (sSSNB vs. cSSNB) at the first and third months, no significant differences were found in any of the ROMs.

- LI and oral analgesia use.

The results obtained in the LI and analgesia, presented descriptively in Table 1, showed the following statistical results.

In the longitudinal study (Table 2) of LI, significant differences were obtained in both treatment groups.

− In patients treated with sSSNB (χ^2^ = 7.58, *p* = 0.023), there were differences between inLI -1mLI (13.50 points [8.75–15.25] vs. 10.50 points [7.75–14.25]) (Z = −1.99; *p* = 0.046) and between inLI -3mLi (13.50 points [8.75–15.25] vs. 6.50 points [10.00–13.00] (Z = −2.51; *p* = 0.012).
− In patients treated with cSSNB (χ^2^ = 18.68, *p* < 0.001), there were differences between inLI -1mLI (12.00 points [10.50–14.25] vs. 6.00 points [3.00–11.25]) (Z = −3.25; *p* = 0.001) and between inLI -3mLI (12.00 points [10.50–14.25] vs. 9.00 points [4.00–11.25]) (Z = −3.07; *p* = 0.002).

In the cross-sectional study (Table 1) between groups (sSSNB vs. cSSNB) at the first and third months, significant differences were only found in 1mLI (10.50 points [7.75–14.25] vs. 6.00 points [3.00–11.25]) (U 48.00; *p* = 0.021) in favor of the cSSNB.

Regarding analgesia intake, longitudinally (Table 2), differences were only found in the group treated with cSSNB (χ^2^ = 11.31, *p* = 0.04), with differences between inAnalgesia – 1mAnalagesia (3.00 points [1.75– 3.00] vs. 2.00 points [0.00–3.00]) (Z–2.43; *p* = 0.015) and in inAnalgesia–3mAnalagesia (3.00 points [1.75–3.00] vs. 2.00 points [0.75–2.25]) (Z = 2.43; *p* = 0.015). The cross-sectional study (Table 1) found no differences in either 1mAnalgesia or 3mAnalgesia.

## 4. Discussion

The introduction of SSNB in PM&R has revolutionized the treatment of chronic shoulder pathology. The use of US as a guide for the management of this interventional technique provides greater precision and safety to these invasive procedures [12]. For this reason, ultrasound-guided SSNB is currently recommended [48].

The typology and combination of drugs with which the SSNB can be carried out harbors a large number of possibilities. [21,23,49]. Currently, the combination of corticosteroids with local anesthetics is a topic of debate, focusing on whether such a combination prolongs the analgesic effects of the blockade. Few studies have addressed this issue in depth, which has ultimately resulted in the absence of a uniform approach to performing SSNB. Recently, a study was published that discussed outcomes in terms of SSNB pain and in its conclusion stated that “While studies suggest efficacy of combined LA and corticosteroid, limitations such as heterogeneity, variability in outcome reporting and short follow-up periods reduce the strength of the evidence” [50].

If we look for studies that compare the results obtained from the use of peripheral blocks with or without corticosteroids, we can find works by authors such as Huynh et al. They concluded in 2015 that the addition of corticosteroids to the local anesthetic solution in peripheral nerve blocks improved postoperative analgesia and reduced the need for additional analgesics in the short term, without significantly impacting side effects [51]. Also, Pehora et al. established in 2017 that the use of dexamethasone in surgeries prolonged short-term effects with low–moderate evidence [25]. And in the same line appears the study by Mohamed et al. in 2025, who reinforced this idea about nerve blocks performed on upper extremities [52]. The study developed by Malhotra et al. proposes performing SSNB but with different doses of corticosteroids [53].

The truth is that most comparative studies focus on short-term results of hours or a few days [25,51,52]. If we want to find works that talk about medium-term results in 2003, we can find the study by EM Shanahan, who studied cSSNB vs. placebo and determined that cSSNB “improves pain, disability and range of motion of the shoulder compared to placebo” with a duration of up to 3 months [54]. And we can also find more recent studies on the effectiveness of cSSNB associated with corticosteroids in the medium to long term. Specifically, some sources estimate that a reduction in shoulder pain symptoms of up to 6 months on average is achieved by applying cSSNB associated with corticosteroids [55]. However, these studies do not make a comparison with a group treated with cSSNB and without associated corticosteroids.

The results of our study need to be broken down and interpreted from two interrelated perspectives: the observed clinical improvement and the significant improvement studied longitudinally and cross-sectionally.

In terms of pain, we established a minimum reduction of 2 VAS points as the MCID [34,35].

VAS scores were comparable between groups, with 8.50 points [7.75–9.25] in sSSNB and 8.00 points [7.75–9.25] in cSSNB.

VAS scores showed significant improvements over time in both treatment groups: sSSNB (χ^2^ = 14.80; *p* = 0.001) and cSSNB (χ^2^ = 19.96; *p* = 0.001). However, only in the cSSNB group did these reductions reach clinical significance, exceeding the pre-established MCID, with decreases of 4 points from baseline, both at month 1 (Z = −3.19; *p* = 0.001) and month 3 (Z = −3.07; *p* = 0.002).

In the between-group analysis, no significant differences were observed in the first month (U = 62.00; *p* = 0.096), although the cSSNB group showed a 2.5-point reduction, greater than the MCID. This finding could be explained by the limited sample size, which could have affected the statistical significance in the first-month comparison. At three months, the cSSNB group did show significantly greater improvement compared to the sSSNB group, with a 3-point reduction on the VAS (U = 53.50; *p* = 0.04).

These results suggest that the use of corticosteroids correlates to greater and sustained reductions in pain.

This improvement in pain by the use of SSNB in association with corticosteroids could be based on the modulation of GABA (inhibitory) receptor expression, influencing the nociceptive activation threshold and producing “neurophysiological changes in the system” [56]. However, it is true that there are also studies that suggest that prolonged use of systemic corticosteroids can produce an increase in excitability and, consequently, pain [57]. In our case, when applied locally and spaced out over time, the use of cSSNB would favor the temporary modulation of GABA receptors without maintaining high levels of systemic corticosteroids and, therefore, without adversely affecting the desired effect.

In terms of functionality, the DASH score required differences greater than 12 points as the threshold accepted as the MCID [58].

Baseline DASH scores were comparable between the sSSNB group with 63.50 points [50.80–80.00] and cSSNB group with 62.60 points [34.30–66.40].

In the longitudinal analysis of DASH, the sSSNB group showed no significant changes over time, whereas the cSSNB group showed significant reductions at follow-up (χ^2^ = 19.74; *p* < 0.001) with differences in scores from baseline of 21.80 points at 1 month (Z = −3.30; *p* = 0.001) and 21.35 points at 3 months (Z = −3.01; *p* = 0.003).

In the between-group analysis, the cSSNB group obtained significantly lower DASH scores than the sSSNB group, both at month 1, with a median difference of approximately 19.20 points (U = 46.00; *p* = 0.017) and at month 3, with a median difference of approximately 12.55 points (U = 43.50; *p* = 0.012). Therefore, both differences exceeded the established MCID, which also confirms clinically important differences.

These results reflect more favorable functionality in the corticosteroid-treated group, similar to what was found with VAS scores.

Very few studies have been found that compare the use or absence of corticosteroids in the medium term and therefore allow for a broad discussion of this topic. Recently, the University of California, Los Angeles, published an article on guidelines and “uses of corticosteroids for chronic pain interventions in adults” [59]. This article recommends performing SSNB with corticosteroids. However, the American Pain Societies that created this guideline make it clear that they recommend their use, as the studies on which these improvements are based have all used this combination and none of them make a comparison like the one in our study [59]. Along the same lines is the meta-analysis by TJ Batten, which concludes that SSNB (using corticosteroids) provides “a safe and effective treatment for shoulder pain” with “significant pain improvement that is maintained beyond 3 months” [60].

When analyzing the ROM data, we must know that the value typically considered the MCID is not determined and that baseline scores for all movements did not initially differ between groups.

That said, the ABD, FLEX and ER movements in those treated with cSSNB showed significant improvements in ROM over time. The ABD showed a median improvement of approximately 42.5° (χ^2^ = 15.10, *p* = 0.001), the Flex ROM 15° (χ^2^ = 13.15; *p* = 0.001) and the ER 5° (χ^2^ = 7.60; *p* = 0.022). However, when comparing each ROM between the two groups (sSSNB vs. cSSNB) at both 1 and 3 months, no significant differences (*p* ≥ 0.05) were found in any of the measurements.

The initial involvement and significant improvement of these movements, especially the ABD, may be related to the fact that the most frequent rotator cuff tendinopathy is that of the SS [61], a key muscle in these movements and to a greater extent in the ABD of the arm [62]. However, it cannot be expressed that there have been differences in the use or non-use of corticosteroids, perhaps also due to the small sample size.

A similar situation occurs when analyzing outcomes in LI. Currently, it is also unknown what values should be considered as MCID, despite it being a widely used tool in pain units and with a significant correlation demonstrated by studies with the VAS. However, of the five items, the analgesia section is the only one that does not demonstrate a relation [63].

However, the scores show clinical improvement, with a reduction in scores compared to inLI and more markedly in the cSSNB group. When analyzing these differences using the Friedman test, both groups (sSSNB and cSSNB) demonstrated this significant improvement compared to inLI. Furthermore, when comparing these measures between groups (sSSNB vs. cSSNB), statistically significant differences were detected only in the first month, with 4.5 points between the medians (U= 48.00; *p* = 0.021) in favor of cSSNB, but this was not the case in 3mLI.

In terms of analgesia use, the only difference between medians was found in the longitudinal analysis of the cSSNB group with differences of 1 point (χ^2^ = 11.31; *p* = 0.04).

It should be noted that no report reflected the appearance of adverse effects associated with the implementation of the SSNB [28,29], such as transient increases in blood pressure, blood glucose levels in diabetic patients, or systemic cardiotoxic events. Similarly, no local phenomena such as hypopigmentation or skin atrophy, nor infections or osteotendinous complications associated with the concomitant use of corticosteroids, were recorded [27]. It is possible that the use of low concentrations of bupivacaine (≤0.25%), combined with the use of US, may have some relation [30]; however, this note should be taken with caution, since the fact that it is not reflected in the report does not mean that it did not occur and since this is a retrospective study, this information may have been lost or not reflected.

### Limitations

During this study, factors susceptible to improvement were identified for future research. Most importantly, the study’s observational and retrospective design requires caution in its conclusions. Proposing prospective studies with triple blinding and randomized treatment assignment would provide greater consistency, external validity and evidence for these results, in addition to allowing for greater follow-up capacity, since it was necessary to extend the age criteria by 10 years due to the difficulty in finding patients who met all the requirements.

It would also be advisable to consider, in the future, the need to further specify shoulder pathology when selecting patients, as very diverse pathologies were reflected in the medical records included under the terms “rotator cuff tendinopathy” or “subacromial syndrome.” In this regard, if the pathology affected the anterior aspect, the SSNB would not be useful since the suprascapular nerve primarily supplies sensation to the entire upper posterior region of the shoulder [17]. Furthermore, the influence of a possible placebo effect or regression to the mean cannot be ruled out, given the fluctuating nature of chronic shoulder pain and the absence of a control group.

The potential for bias in these studies is high, so measures to avoid it were taken into account. Homogeneity among the samples obtained was confirmed to avoid selection bias. To this end, we compared systematically baseline characteristics (age, gender, comorbidities, VAS, DASH, ROM and IL) between the two groups. The analysis showed no statistically significant differences in any of these variables (*p* > 0.05), supporting the homogeneity of the sample in all cases. Despite the homogeneity and normality of the tests, it was decided to use non-parametric tests given the sample size of each group. The main scales selected were those commonly known as VAS and DASH to avoid classification and observer bias. Despite all this, the limited sample size could have reduced the effective statistical power and increased the risk of type II error.

Patients with “missing data” were eliminated from the study. However, a very important fact, which adds to the retrospective nature of this study, was that patients who appeared to offer promising results in the first month of the SSNB did not attend their follow-up appointment in the third month. This could translate into a biased view due to loss of follow-up bias when assessing the real comparative efficacy of both treatments.

## 5. Conclusions

The cSSNB block produced a clinically significant and more sustained improvement in pain and function compared to the sSSNB block.

Significant improvements in ABD, FLEX and ER were observed over time in patients treated with cSSNB, but no differences were observed between the two types of blocks.

Both blocks significantly improved LI, with the most marked improvement observed with the cSSNB at one month. The reduction in analgesia was only significant longitudinally with the cSSNB, with no differences between the blocks.

Therefore, the results of our study suggest that the combined use of corticosteroids in SSNB appears to be associated with better short- to medium-term outcomes in terms of pain and function, compared with the use of SSNB without corticosteroids in chronic rotator cuff conditions. This warrants further prospective validation.

## Figures and Tables

**Table 1:** Descriptive results and comparative study between groups (sSSNB vs. cSSNB) of the medians of the main study variables in a cross-sectional manner at the beginning, at one month and at three months. PT: Physical therapy, PRP: platelet-rich plasma. * Significant results. ° Degrees.

Variable		sSSNB	cSSNB	Value (Test)	*p*
**Age,** years		61.00 [53.0–66.00]	63.00 [58.50–70.00]	74.50 (U)	0.279
**Gender**		Male: 3 (21.4%) Female: 11 (78.6%)	Male: 3 (21.4%) Female:11(78.6%)	- (Fisher exact)	1
**Comorbidities**		S/N: 7 (50%) HTA: 5 (35.7%) DM: 2 (14.3%)	S/N: 6 (42.9%) HTA: 5 (35.7%) DM: 3 (21.4%)	0.27 (χ^2^ de Pearson)	0.871
**Previous treatments**		nothing: 5 (35.7%) Infiltrations: 7 (50%) SSNB: 1 (7.1%) PT: 1 (7.1%)	nothing: 4 (28.6%) Infiltrations: 8 (57.1%) SSNB: 1 (7.1%) PRP: 1 (7.1%)	2.18 (χ^2^ de Pearson)	0.703
**VAS,** points	**In**	8.50 [7.75–9.25]	8.00 [7.75–9.25]	95.50 (U)	0.906
	**1m**	6.50 [3.75–8.00]	4.00 [2.75–6.25]	62.00 (U)	0.096
	**3m**	7.00 [5.00–8.25]	4.00 [2.00–7.00]	53.50 (U)	0.040 *
**DASH,** points	**In**	63.50 [50.75–80.00]	62.6 [34.25–66.43]	81.00 (U)	0.434
	**1m**	60.00 [47.50–71.56]	40.80 [23.95–58.33]	46.00 (U)	0.017 *
	**3m**	53.80 [44.18–68.58]	41.25 [26.83–50.71]	43.50 (U)	0.012 *
**FLEX** °	**In**	150.00 [97.50–172.50]	165.00 [90.00–180.00]	94.00 (U)	0.851
	**1m**	170. 00 [137.50–180.00]	180.00 [150.00–1800]	80.00 (U)	0.382
	**3m**	170.50 [107.50–180.00]	180.00 [157.50–180.00]	72.00 (U)	0.194
**ABD** °	**In**	125.00 [90.00–180.00]	137.50 [90.00–180.00]	92.50 (U)	0.794
	**1m**	155.00 [100.00–180.00]	180.00 [131.25–180.00]	71.50 (U)	0.199
	**3m**	150.00 [107.50–180.00]	180.00 [155.00–180.00]	67.00 (U)	0.114
**IR** °	**In**	90.00 [77.50–90.00]	90.00 [75.00–90.00]	96.50 (U)	0.931
	**1m**	90.00 [77.50–90.00]	90.00 [85.00–90.00]	92.50 (U)	0.739
	**3m**	90.00 [70.00–90.00]	90.00 [90.00–90.00]	71.50 (U)	0.089
**ER** °	**In**	90.00 [72.50–90.00]	85.00 [43.75–90.00]	78.50 (U)	0.309
	**1m**	90.00 [77.50–90.00]	90.00 [50.00–90.00]	80.50 (U)	0.348
	**3m**	90.00 [70.00–90.00]	90.00 [72.50–90.00]	94.00 (U)	0.825
**LI,** points	**In**	13.50 [8.75–15.25]	12.00 [10.50–14.25]	82.00 (U)	0.459
	**1m**	10.50 [7.75–14.25]	6.00 [3.00–11.25]	48.00 (U)	0.021 *
	**3m**	6.50 [10.00–13.00]	9.00 [4.00–11.25]	83.00 (U)	0.488
**Analgesia,** points	**In**	3.00 [1.75–3.00]	3.00 [1.75–3.00]	94.00 (U)	0.845
	**1m**	1.50 [1.00–3.00]	2.00 [0.00–3.00]	91.00 (U)	0.742
	**3m**	2.50 [0.75–3.00]	2.00 [0.75–2.25]	86.00 (U)	0.573

**Table 2:** Longitudinal statistical results using Friedman and Wilcoxon tests, stratified by groups. * Significant results. Post hoc test results show exploratory data.

Variable	Group	Friedman’s χ^2^	*p* Value	Post Hoc Tests. (Wilcoxon, *p* Value)		
				Initial vs. 1 Month	Initial vs. 3 Months	1 Month vs. 3 Months
**VAS**	**sSSNB**	14.80	0.001 *	Z = −2.95, *p* = 0.003 *	Z = −2.85, *p* = 0.004 *	Z = −1.27, *p* = 0.205
	**cSSNB**	19.96	0.001 *	Z = −3.19, *p* = 0.001 *	Z = −3.07, *p* = 0.002 *	Z = −0.42, *p* = 0.676
**DASH**	**sSSNB**	1.33	0.513	-	-	-
	**cSSNB**	19.74	<0.001 *	Z = −3.30, *p* = 0.001 *	Z = −3.01, *p* = 0.003 *	Z = −0.801, *p* = 0.423
**FLEX**	**sSSNB**	4.07	0.131	-	-	-
	**cSSNB**	13.15	0.001 *	Z = −2.38, *p* = 0.018 *	Z = −2.53, *p* = 0.012 *	Z = −1.07, *p* = 0.285
**ABD**	**sSSNB**	0.48	0.786	-	-	-
	**cSSNB**	15.10	0.001 *	Z = −2.53, *p* = 0.011 *	Z = −2.68, *p* = 0.007 *	Z = −1.07, *p* = 0.285
**IR**	**sSSNB**	0.33	0.846	-	-	-
	**cSSNB**	4.769	0.92	-	-	-
**ER**	**sSSNB**	1.20	0.549	-	-	-
	**cSSNB**	7.60	0.022 *	Z = −1.63, *p* = 0.102	Z = −2.03, *p* = 0.042	Z = −1.34, *p* = 0.180
**LI**	**sSSNB**	7.59	0.023 *	Z = −1.99, *p* = 0.046 *	Z = −2.51, *p* = 0.012 *	Z = −1.41, *p* = 0.159
	**cSSNB**	18.67	<0.001 *	Z = −3.25, *p* = 0.001 *	Z = −3.07, *p* = 0.002 *	Z = −1.54, *p* = 0.123
**Analgesia**	**sSSNB**	2.00	0.368	-	-	-
	**cSSNB**	11.31	0.004 *	Z = −2.42, *p* = 0.015 *	Z = −2.43, *p* = 0.015 *	Z = 0.00, *p* = 1.000

## Data Availability

The data presented in this study are available on request from the corresponding author due to legal issues related to patient data privacy.

## Abbreviations

The following abbreviations are used in this manuscript:

ROM	Range of Motion
SSNB	Suprascapular Nerve Block
US	Ultrasound
DASH	Disabilities of the Arm, Shoulder and Hand
VAS	Visual Analog Scale
LI	Lattinen Index
PGI-I	Patient Global Impression of Improvement
CGI-GI	Clinical Global Impression of Global Improvement
FLEX	Flexion
ABD	Abduction
ER	External Rotation
IR	Internal Rotation
PT	Physical Therapy
PRP	Platelet-Rich Plasma
SS	Supraspinatus
HURS	Hospital Universitario Reina Sofia of Cordoba
PM&R	Physical Medicine and Rehabilitation
MCID	Minimal Clinically Important Difference
DAG	Directed Acyclic Graph
